# Extraskeletal myxoid chondrosarcoma in the lung: asymptomatic lung mass with severe anemia

**DOI:** 10.1186/1746-1596-7-112

**Published:** 2012-08-28

**Authors:** Qianjun Zhou, Guangzhong Lu, Aiqun Liu, Tadasu Kohno

**Affiliations:** 1 Department of General thoracic surgery, Shanghai First People's Hospital, Shanghai Jiao Tong University School of Medicine, Shanghai, 200080, P. R. China; 2 Department of Pathology, Shanghai First People's Hospital, Shanghai Jiao Tong University School of Medicine, Shanghai, 200080, P. R. China; 3 Department of Radiology, Shanghai First People’s Hospital, Shanghai Jiao Tong University School of Medicine, Shanghai, 200080, P. R. China; 4 Department of General thoracic surgery, Toranomon Hospital, Tokyo, 1058470, Japan; 5 Department of Microbiology and Immunology, State university of New York at Buffalo, Buffalo, NY, 14214, USA

**Keywords:** Extraskeletal myxoid chondrosarcoma, Lung, Immunohistochemistry, Anemia

## Abstract

Extraskeletal myxoid chondrosarcoma (EMC) is a rare soft-tissue sarcoma, which primarily occurs deep in the extremities, especially in skeletal muscle, or tendon. EMC of the pleura has been described, however, no case of primary EMC arising from lung has been previously reported. We describe herein, a 51-year-old Asian female initially manifested with signs of severe anemia who presented with a lung mass unrelated to pleura that was morphologically typical EMC, with strong immunoreactivity for vimentin and NSE. Two weeks after resection, the anemia was cured. The patient continued with follow-up, without sign of abnormality 32 months after operation.

**Virtual slides:**

The virtual slides for this article can be found here: http://www.diagnosticpathology.diagnomx.eu/vs/2882199847396682

## Background

Extraskeletal myxoid chondrosarcoma (EMC) was first reported by Enzinger and Shiraki in 1972 as a rare soft-tissue sarcoma, which primarily occurs deep in the extremities, especially in skeletal muscle, or tendon [1]. Unusual locations include tongue, retroperitoneum, spine, intracranium, testis, inguinal region, synovium, mammary gland, and labium.

We report the clinicopathological features of a female patient with EMC of the lung initially manifested with asymptomatic lung mass with severe anemia determined by the evidence of histological and immunohistochemical examination.

## Case presentation

**Figure 1:**
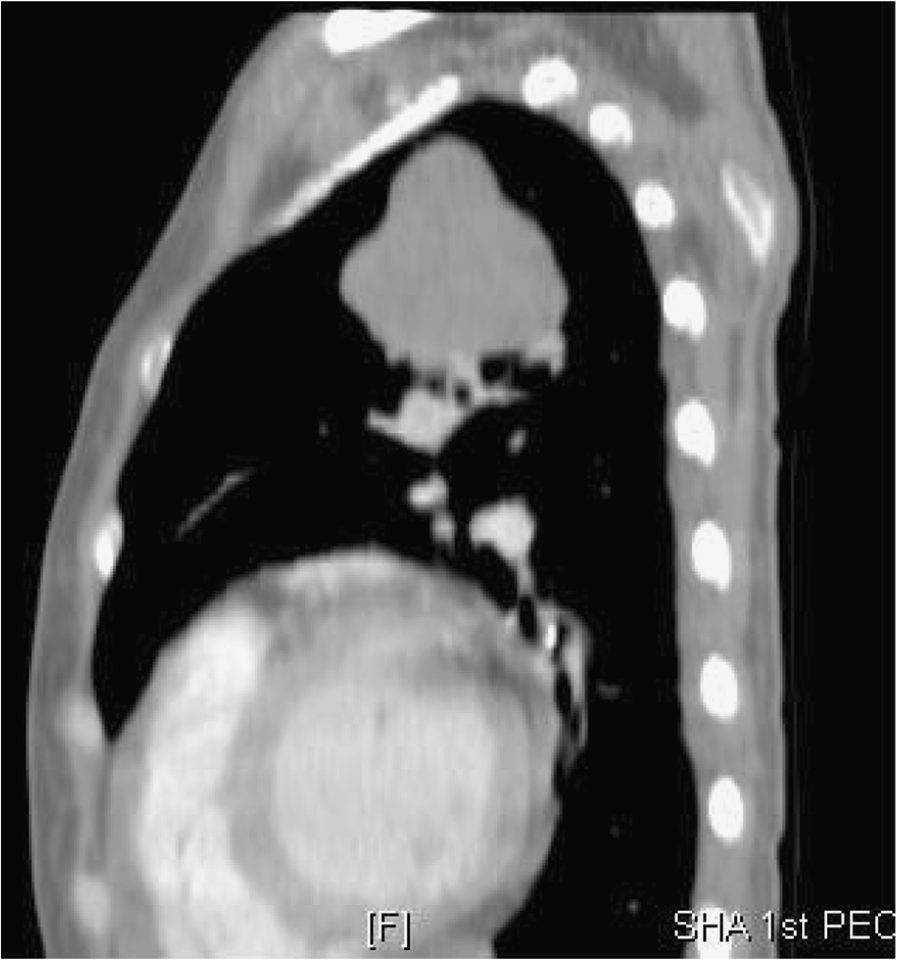
Preoperative radiological features: large mass involving the left upper lobe.

A 51-year-old Asian woman, who was a social worker, nonsmoker, was admitted to the Department of Hematology in August 2009 because of severe anemia and emaciation without cough, sputum, and hemoptysis. Physical examination revealed marked sign of anemia but failed to find any masses in the soft tissue of extremities or pelvis through palpation. A computed tomography (CT) scan disclosed a 41 × 33 mm parenchymal mass on apico-posterior segment of left upper lobe, CT value was 26 Hounsfield unit (HU). The patient had a 10-year history of left lung nodule by chest x-ray, however, no films were available currently. Whole blood cell count was characterized as microcytic hypochromic anemia, with RBC 2.67 × 10^12^/L, Hb 54 g/L. Serum iron was 2.2 μmol/L, total iron binding capacity 74 μmol/L. Serum IgG 18.3 g/L, and a serum protein electrophoresis examination found no monoclonal protein. Tumor markers such as carcinoembryonic antigen (CEA) and neuron specific enolase (NSE) were all negative. Bone marrow smear and biopsy showed normocellularity with lower intracellular iron. Bronchoscopic examination with biopsy, brushing and washing found no abnormality. A bone scan was negative for other lesions. There were no metastatic signs in brain and abdomen by CT scan. After the therapy of iron supplement and red blood cell transfusion, Hb was slightly elevated. The patient was transferred for surgery on October, and thoracic CT scan showed the tumor was 55 × 48 mm, with CT value 34 HU, 54 HU in contrast enhancement scanning (Figure 1). After anemia had been cured by transfusion, left exploratory thoracotomy was planned. Operation revealed a peripheral pulmonary mass on left upper lobe without pleural indentation or swollen lymph node in hilum and mediastinum, and the patient underwent left upper lobectomy with systemic lymph node dissection. The patient recovered smoothly and was discharged in a good condition without anemia after two weeks. Follow-up continued every three month after operation by chest CT scan and CBC. There was no sign of abnormality 32 months after operation.

Grossly, the left upper lobe measured 20 cm × 10.5 cm × 4 cm, and the lesion was located in lung parenchyma 2 cm away from the resection margin of left upper bronchus. On cut surface, the tumor measured 5 cm × 5 cm × 3.2 cm in size, pale gray, soft, well demarcated, lobulated with a gelatinous texture.

**Figure 2:**
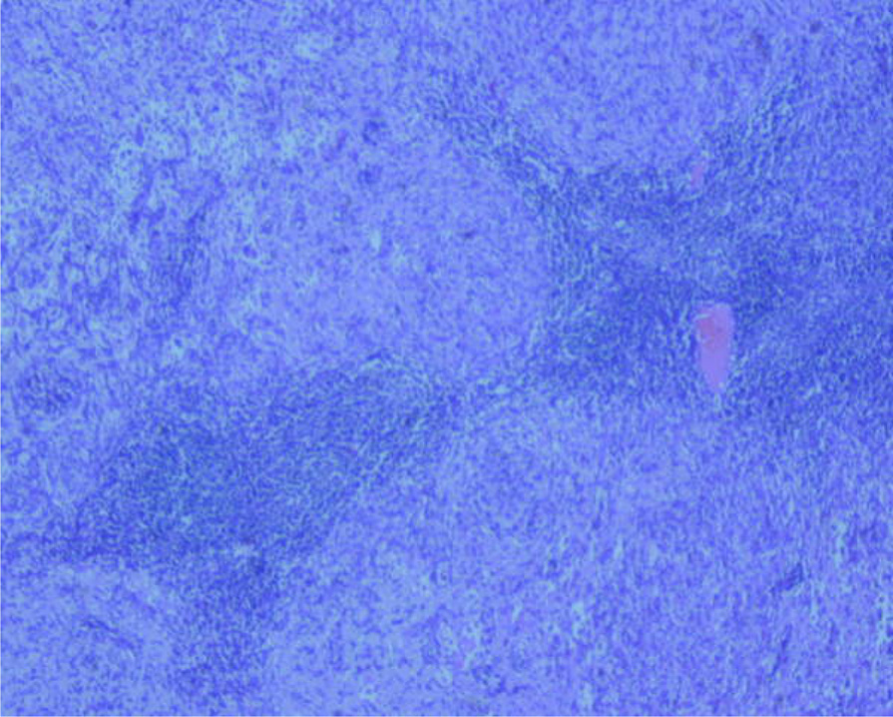
Low magnification shows the well-circumscribed tumor with multinodular architecture. ×40.

**Figure 3:**
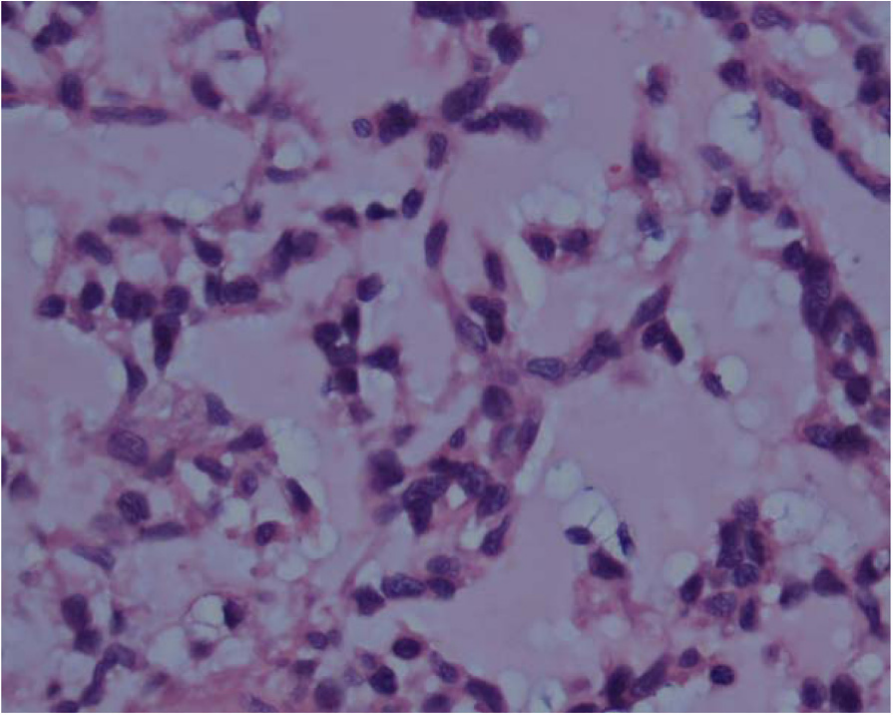
High power image of spindle cells in cords present within a myxoid background. The cells are vaculoated showing nuclear atypia ×400.

**Figure 4:**
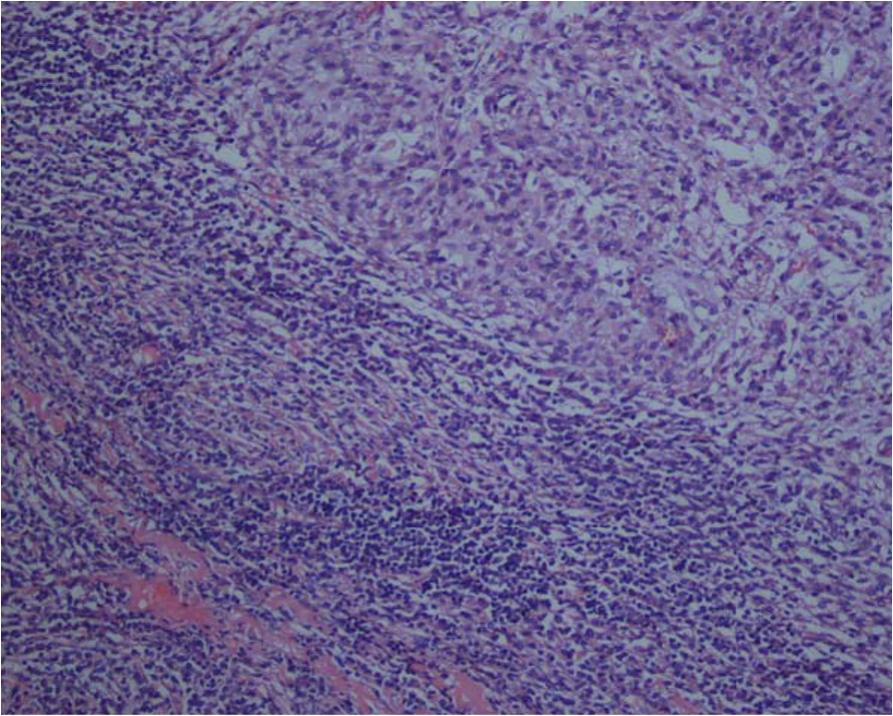
Medium power image illustrates the plasma cells and lymphocytes surrounding the tumor.

Microscopic examination showed the characteristic features of EMC, including a well-circumscribed, multilobulated architecture separated by incomplete fibrous septae, relatively uniform oval to short spindle shaped cells arranged in short anastomosing strands and cords embedded in abundant myxoid matrix (Figure 2). The cells were characterized by hyperchromatic nucleus and tiny nucleolus, some of which showed grooves (Figure 3). There were a large number of plasma cells and a few lymphocytes in fibrous septae to form the compact zone (Figure 4). No hyaline cartilage was seen. Mitotic figures were 1/50HPF. No infiltration into adjacent lung tissue was seen.

**Figure 5:**
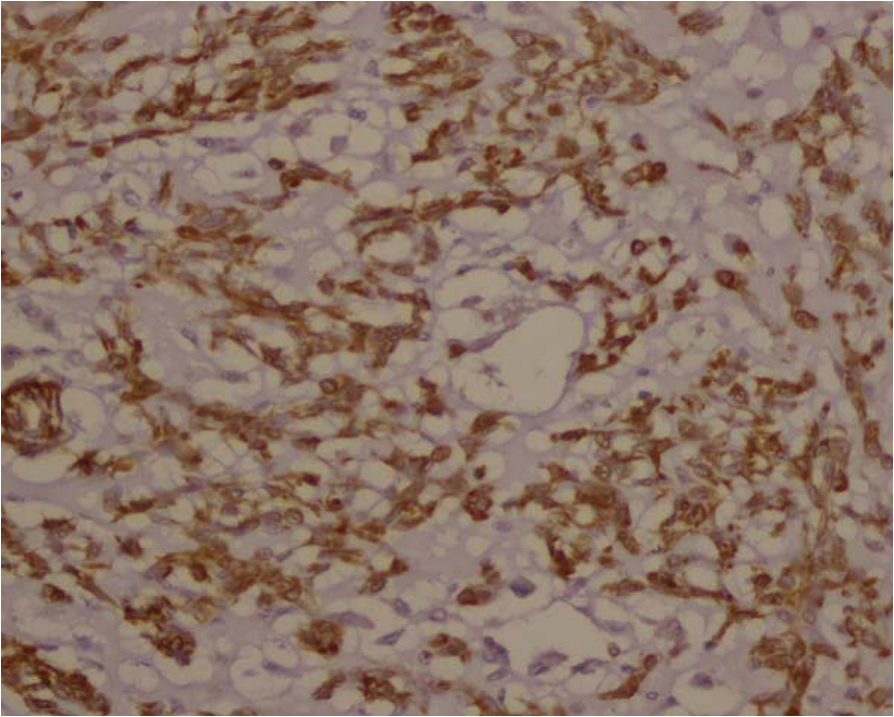
Photomicrographs shows the strong expression of Vimentin in tumor cells.

**Figure 6:**
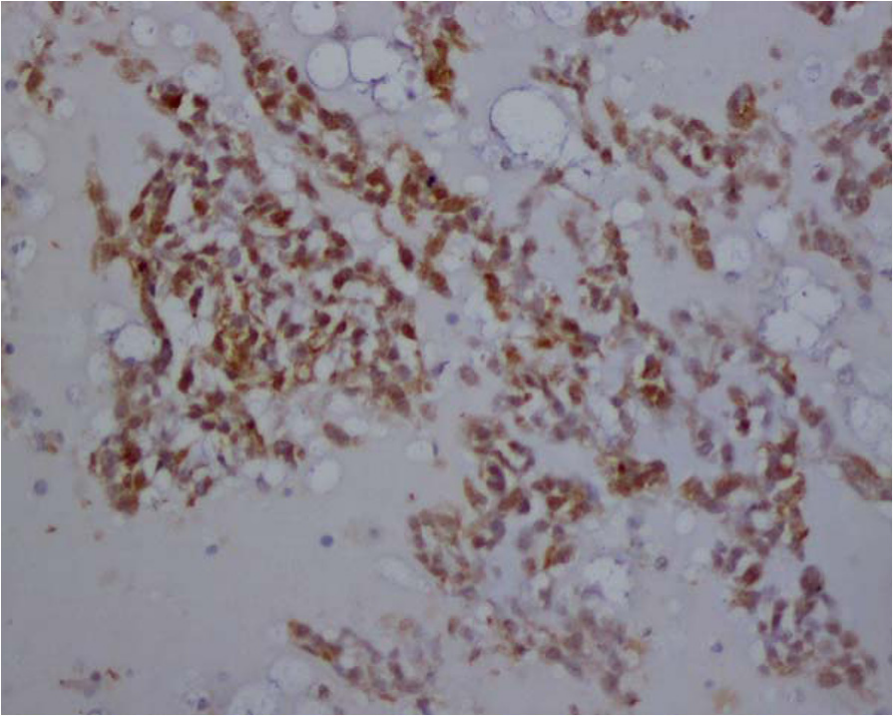
The tumors are strongly immunopositve to neuron-specific enolase.

**Figure 7:**
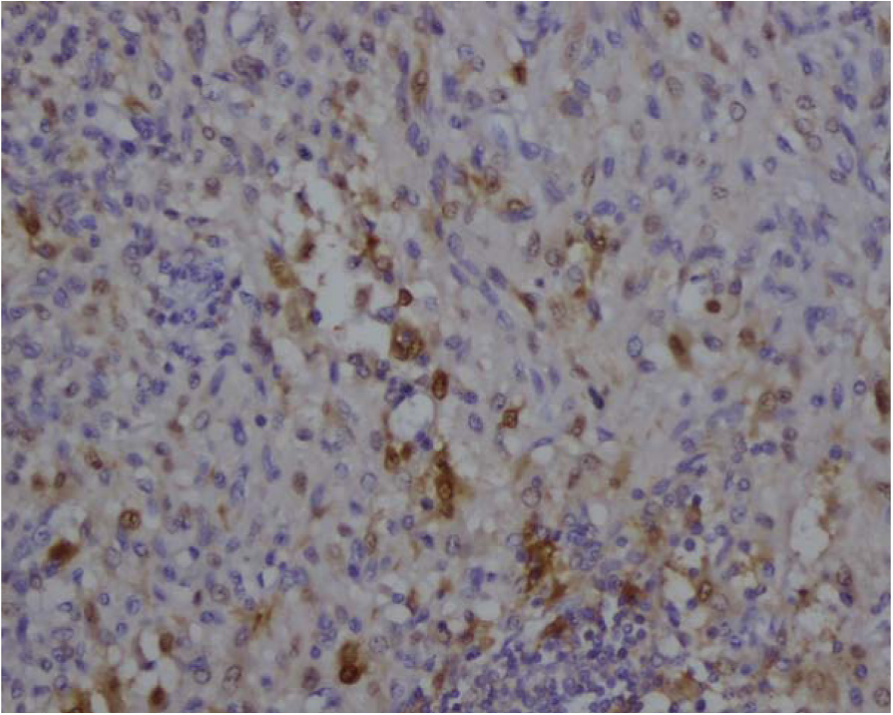
S-100 protein expression was weak and focal in the tumor.

Special stains revealed glycogen in the cytoplasm of the tumor cells, while the surrounding extracellular matrix was positive with Alcian blue stain. By immunohistochemical study, vimentin and NSE were strongly expressed in the tumor cells (Figure 5, 6), while S-100 protein was weak and focal (Figure 7). The other markers including cytokeratin, epithelial membrane antigen (EMA), P63, smooth muscle actin (SMA), muscle specific actin (MSA), desmin, KP1, calretinin were all negative. IFN-β was immunopositive in the tumor cells.

## Discussion

EMC has mainly been reported in the areas of orthopedics, neurosurgery, and otolaryngology [1]. As with our case, the characteristic features of well-circumscribed, multilobulated configuration with incomplete fibrous septae, oval to spindle shaped cells arranged in short anastomosing strands embedded in abundant myxoid matrix and typical histochemical findings favored the diagnosis of EMC [2]. Since the lesion located at the periphery of the lung, fine needle aspiration biopsy (FNAB) should be initially done to help diagnosis. Although cytologic characteristics are not decisive for diagnosis, the presence of intranuclear cytoplasmic inclusions, grooves and cytoplasmic vacuolization could be suggestive to EMC diagnosis [3]. S-100 protein was initially found in most EMC, but recent studies proved that less than 20 % of EMC are S-100 positive [4]. In some cases of EMC, there is neuroendocrine differentiation [5]. A specific chromosomal translocation t(9;22)(q22;q12) leading to a fusion gene, EWS-CHN has been found in 75 % EMC cases [6], and another fusion gene TAF2N-CHN caused by t(9;17)(q22;q11.2) translocation has recently been identified [5]. Although primaries should not have difficulty in diagnosis for experienced pathologists, the morphologic differential diagnosis in our case are parachordoma, chordoma, soft tissue chondroma, extraskeletal mesenchymal chondrosarcoma,as well as pulmonary metastases of EMC. Parachordoma is an indolent soft tissue tumor predominantly in the extremities and trunk, occasionally in the chest wall. The diagnosis is excluded because there were no typical histopathologic and immunohistochemical features of parachordoma such as plasma cell-like appearance of the tumor cells with chondrometaplasia myoepithelium, and expression of cytokeratin, and EMA. Chordoma appears as lobulated tumors, with cord pattern tumor cells scattering within abundant myxoid stroma. The diagnosis is ruled out by a lack of physaliferous cells, and no expression of cytokeratin. Soft tissue chondroma composed of small cells with hyaline cartilage lobule which lies near the joints of extremities, and S-100 is uniquely positive. Extraskeletal mesenchymal chondrosarcoma arising in lung [7], chest wall, pleural and mediastinum has been reported. The lack of small undifferentiated cells surrounding narrow vascular spaces in a haemangiopericytoid (haemangiopericytoma-like) pattern with mature cartilage island would exclude extraskeletal mesenchymal chondrosarcoma. As lung is the most frequent site of metastases in EMC, it is necessary to differetiate primary EMC in lung from lung metastases of EMC. However, it was not difficult to exclude lung metastases of EMC originated in the other parts of the body after comprehensive clinical evaluation and CT scan, with particular attention to the distal parts of the extremities. There was not any evidence of tumor in the body other than in the lung of the patient. Extraosseous (extramedullary) plasmacytoma (EMP) is defined as localized plasma cell neoplasms arising in tissues other than bone, and EMP in lung has been reported [8]. In our case, there are abundant plasma cells and aggregated lymphocytes in tumor fibrous septae and adjacent lung tissue which might be the anti-tumor reaction of immune system. As to EMP, the neoplastic cells are similar to normal plasma cells with very few lymphocytes. Last, our case should also be differentiated from primary pulmonary sarcomas. Pulmonary myxoid liposarcoma is multinodular-architecture in myxoid stroma with Vimentin and S-100 positive which is similar to EMC, however, lack of lipoblasts and plexiform capillary network exclude the diagnosis [9]. Immunostains can be helpful to rule out rhabdomyosarcoma, leiomyosarcoma. Primary and metastatic chondrosarcoma of lung have been documented, and the multilobular neoplasm with chondroid and myxoid matrix which is positive to Vimentin and S-100, negative for epithelial markers might cause the confusion with EMC [10]. In this case, absence of predominant chondromatous lesion helps to exclude chondrosarcoma. Pulmonary carcinosarcoma is ruled out for lack of carcinomatous component [11].

Although EMC is most common in the soft tissues of the extremities, in fact, the primary site can be anywhere in the body [1]. To our knowledge, no case of EMC arising in lung has been reported to date. Primary EMC of the pleura has been described by Goetz et al. [12]. From the CT scan, a parenchymal mass of left upper lobe can be found with distinct margin from the intact visceral pleura, which were also proved by microscopic examination on cut section of tumor. We suggest that the origin might be primitive mesenchymal cell, or associated with the tracheobronchial cartilage [13,14].

Anemia is one of the characteristics in this patient which has not been reported previously in EMCs. Patients afflicted with malignancy often develop anemia, and some cytokines, particularly TNF-α,TGF-β,IFN-β, IL-1,are found increasing in many malignant diseases [15,16]. The function of these cytokines is related to retention of iron in the reticuloendothelial system, gastrointestinal tract and liver and exert inhibitory effects on erythroid precursors [17]. As in our case, expression of IFN-β in tumor cells, significant in excessive type IFN signaling, inhibits erythropoiesis through decreased Bcl-XL expression level and enhanced apoptosis of erythroblasts [18]. Also, negative bone marrow cytology rules out occurrence of primary hematological malignancies, such as multiple myeloma. It is intriguing that the anemia was corrected just after the removal of the tumor, suggesting that the cause of anemia relies on the tumor itself.

EMC shows a tendency to affect patients beyond their fifth decade [19]. EMC was initially viewed as a low-grade sarcoma with prolonged and indolent clinical course, but recent studies found its unfavorable prognosis with high rate of recurrence and metastases [20]. Significant in the past history was that our 51-year-old patient had a left lung nodule examined by routine chest x-ray check for around 10 years, which was in accordance with those previous reports. Since EMC has a high rate of recurrence and metastases, and the death caused by tumor often occurs in a long postoperative period, sometimes more than 10 years after diagnosis, so we suggest that frequent and long-term follow up is needed for our patient.

## Conclusion

Our case is the first case of EMC located in the lung. It highlights the possibility and difficulty of diagnosis of this rare tumor in the lung, especially in the patients with history of long-term and slowly-growing lung nodule. Characteristic histological features supported by immunohistochemical reactivities, help to diagnose EMC of the lung, although it is exceedingly rare in this location.

## Consent

Written informed consent was obtained from the patient’s relatives for publication of this case report and any accompanying images.

## Competing interests

The authors declare that they have no competing interests.

## Authors’ contributions

QZ and GL equally contributed to this article. QZ, GL and KT conceived and designed the study, QZ and GL wrote the manuscript. QZ and AL participated in patient’s management. GL made the pathological explorations. All authors read and approved the final manuscript.
